# Home-based HIV testing for men preferred over clinic-based testing by pregnant women and their male partners, a nested cross-sectional study

**DOI:** 10.1186/s12879-015-1053-2

**Published:** 2015-07-30

**Authors:** Alfred Onyango Osoti, Grace John-Stewart, James Njogu Kiarie, Richardson Barbra, John Kinuthia, Daisy Krakowiak, Carey Farquhar

**Affiliations:** Department of Obstetrics & Gynecology, University of Nairobi, Nairobi, Kenya; AIC Kijabe Hospital, 20, Kijabe, Kijabe, 00220 Kenya; Department of Epidemiology, University of Washington, Seattle, USA; Department of Global Health, University of Washington, Seattle, USA; Department of Medicine, University of Washington, Seattle, USA; Department of Biostatistics, University of Washington, Seattle, USA; Vaccine and Infectious Disease Division, Fred Hutchinson Cancer Research Center, Seattle, USA; Departments of Research, Obstetrics & Gynecology, Kenyatta National Hospital, Nairobi, Kenya

**Keywords:** Male, Partner, Couple, HIV testing, Pregnancy, Antenatal settings, Home-based

## Abstract

**Background:**

Male partner HIV testing and counseling (HTC) is associated with enhanced uptake of prevention of mother-to-child HIV transmission (PMTCT), yet male HTC during pregnancy remains low. Identifying settings preferred by pregnant women and their male partners may improve male involvement in PMTCT.

**Methods:**

Participants in a randomized clinical trial (NCT01620073) to improve male partner HTC were interviewed to determine whether the preferred male partner HTC setting was the home, antenatal care (ANC) clinic or VCT center. In this nested cross sectional study, responses were evaluated at baseline and after 6 weeks. Differences between the two time points were compared using McNemar’s test and correlates of preference were determined using logistic regression.

**Results:**

Among 300 pregnant female participants, 54 % preferred home over ANC clinic testing (34.0 %) or VCT center (12.0 %). Among 188 male partners, 68 % preferred home-based HTC to antenatal clinic (19 %) or VCT (13 %). Men who desired more children and women who had less than secondary education or daily income < $2 USD were more likely to prefer home-based over other settings (*p* < 0.05 for all comparisons). At 6 weeks, the majority of male (81 %) and female (65 %) participants recommended home over alternative HTC venues. Adjusting for whether or not the partner was tested during follow-up did not significantly alter preferences.

**Conclusions:**

Pregnant women and their male partners preferred home-based compared to clinic or VCT-center based male partner HTC. Home-based HTC during pregnancy appears acceptable and may improve male testing and involvement in PMTCT.

## Introduction

Male partner involvement in prevention of mother-to-child HIV transmission (PMTCT) has been associated with enhanced uptake of PMTCT interventions and infant HIV-free survival [1, 2]. Male partner HIV testing is a critical link to male involvement in PMTCT and other aspects of reproductive health for sexually active couples in stable relationships. For example, male home-based HIV testing and counseling (HTC) has been associated with decreases in HIV related high-risk sexual behaviors [3, 4]. However, despite the availability of multiple options, including mobile and fixed voluntary counseling and testing (VCT) centers, antenatal care (ANC) clinic and home-based HTC, less than one-third of male partners of pregnant women undergo individual or couple-based HTC in sub-Saharan Africa [5–8].

Uptake of male partner HTC during the antenatal period is low [7, 9, 10]. Current options for HTC include VCT center-based testing, antenatal care (ANC) clinic-based testing, or home based testing (HBHCT). As a client-initiated approach, VCT center-based testing may face barriers due to stigma and vulnerability because of attitudes or assumptions regarding individuals who request HIV testing. Individuals and couples may perceive seeking VCT testing as an indication of high-risk sexual behavior. These barriers may be lowered in routinized HIV testing models, such as provider-initiated testing and counseling (PITC) that is administered to all individuals seeking care [11]. VCT center testing may not improve disclosure of HIV results since the testers are rarely counseled and tested as couples [12]. ANC clinic-based male partner HTC has remained low despite improvisations like providing male partner invitation letters or efforts to make ANC settings male-friendly [7, 10, 13]. Furthermore, while ANC male testing provides an opportunity for couple HTC, most men do not accompany their pregnant partners to ANC because this is considered a female domain [14]. Pregnant women and their male partners also cite facility space limitations and staff attitudes as barriers to male partner testing in the ANC clinic [15, 16].

Home-based HTC (HBHTC) has consistently had high acceptability (>70 %), particularly among individuals in stable relationships [17, 18]. Provider-initiated home-based HTC has been shown to increase HTC of families and partners of HIV-infected individuals [19]. In our recent randomized trial that compared home versus clinic-based male partner testing during pregnancy in Kenya, there was over a 2-fold increase in male partner access and more men underwent HTC at home (85 %) than in the clinic (36 %) [20]. Home testing may be advantageous as it enables facilitated disclosure of HIV results and increases access to male partner for couple testing, which are important concerns of women who undergo individual HTC during pregnancy [21]. Despite the success of home-based testing in a few research studies in reaching male partners, this method has not become standard practice for antenatal care. It is therefore important to determine preferences of men for HTC venue, so as to inform programs regarding where to focus male HIV testing resources and achieve greatest impact.

In order to determine the most acceptable setting for male partner HTC, we interviewed participants in a randomized trial on their preferred setting for male partner HTC, either home-based, VCT center-based or ANC clinic-based HTC. We compared setting preference at baseline and at a 6-week follow-up visit and evaluated the preferred male partner testing settings among participants randomized to either clinic-based or home-based testing.

## Methods

### Study design and setting

This was a cross-sectional study nested within a randomized clinical trial conducted in a rural high HIV-prevalence setting in Nyanza province, Kenya at the Ahero Sub-district Hospital. Participating couples were followed up six weeks after enrollment.

### Subject selection

Study participants were pregnant women and their male partners. Women were screened during their first antenatal care (ANC) clinic visit and randomized to either a home visit with their male partner or an invitation to bring their male partner to the ANC clinic for couple HTC. Male partners were reached during either home or clinic visit, as described elsewhere [20]. Women were 18 years or older, in stable relationships, unaccompanied by their male partners during the ANC clinic visit and had not received couple HTC in the index pregnancy, prior to this first ANC visit. HIV-positive women were eligible if they had not previously disclosed their HIV status to their male partners. Male participants were 18 years or older partners of participating pregnant women.

### Study procedures

Following informed consent and enrollment, trained community health workers, who were also experienced HIV counselors, interviewed participants in both study arms using Audio Computer Assisted Self Interview (ACASI) on a Samsung® Google Nexus smartphone. After completing the antenatal procedures, a scheduled 6-week follow-up visit was conducted either at home or at the clinic during which an exit ACASI was also administered. The ACASI were conducted privately either in a room at the clinic or within the participant homes in either English or Dholuo (the local dialect). Participants were interviewed on a variety of topics, including preferred setting of male testing, reproductive health decisions, HIV risk factors and uptake of HIV prevention interventions. To determine the type of preferred testing, participants were asked at baseline and follow-up if they favored either home-based male partner testing, antenatal clinic-based male partner testing or VCT center-based testing. ACASI data were collected on an Open Data Kit (ODK) platform.

### Sample size and study outcomes

The randomized trial was powered to detect a 50 % increase in male partner tracing and uptake of couple HTC between the clinic and home-based arms. This secondary analysis included all study participants who were interviewed both at baseline and at follow-up. The primary study outcome was the preferred setting of male HTC at baseline and follow-up. Secondary outcomes were correlates of each male HTC setting stratified by gender and couple HTC status and changes in preferred male HTC setting between enrolment and the 6-week follow-up visit.

### Statistical analysis

Smartphone ACASI data were saved on ODK Collect, and imported into Stata® 12, which was used for analyses. We evaluated the proportions of participant preference for each of the three settings of male partner testing during enrollment and follow-up stratified by gender. Chi-squared tests and 95 % confidence intervals were used to test for significant differences between the different settings at baseline and follow-up. Logistic regression analyses were conducted to identify correlates of setting preference for each gender and to adjust for partner testing status. McNemar’s test was used to compare changes in setting preferences between baseline and follow-up.

### Ethical statement

The Institutional Review Boards at University of Washington, University of Nairobi and Kenyatta National Hospital approved the study protocol. All study participants provided written informed consent in English or Dholuo. The community advisory board provided oversight and advice. The parent study protocol was registered at ClinicalTrials.gov, registration number, NCT01620073.

## Results

### Sociodemographic characteristics

**Fig. 1:**
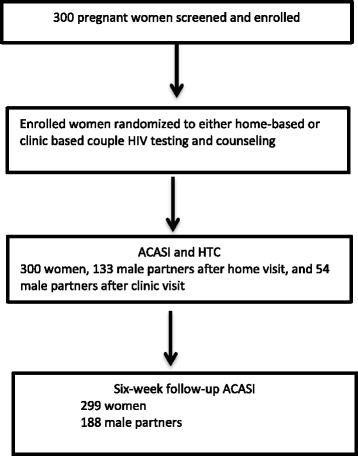
Enrollment and follow-up of pregnant women and their male partners

Between July 2012 and February 2013, 300 eligible pregnant women were randomized and assigned to home-based (*n* = 150) or clinic-based (*n* = 150) male partner HTC. One hundred and eighty-eight male partners of these women were enrolled either at home (*n* = 133, 70.7 %) or antenatal care (ANC) clinic (*n* = 55, 29.3 %) (Fig. 1). This difference in male enrollment reflects the higher effectiveness of the HBHTC strategy in this trial.

Participating men had a median age of 29 years (interquartile range [IQR] 25, 35) and 42 % of these men had secondary or higher level of education. Most men (87.8 %) were in monogamous marital relationships. The majority of men (60.1 %) reported a daily household income of > $2 USD. Thirty-one (17.0 %) men reported concurrency, while 20 (10.6 %) men perceived their female partners as having concurrent partnerships. During the preceding six months, 34 (18.1 %) men reported experiencing physical threat from their female partner while 28 (14.9 %) received threats from other partner(s) or family member. Few men reported experiencing forced sex over the preceding six months either from their current sex partner (2.7 %) or another partner or family member (1.1 %). HIV prevalence among the participating male partners was 16 %. Most men reported that their female partners were multiparous (59 %) and desired more children (84.0 %). About two-thirds of men (68.1 %) reported no contraceptive use by their partner prior to the index pregnancy.

The baseline characteristics of women enrolled in the primary study have previously been described and are summarized here (Table 1) [20]. Compared to male partners, women were younger with a median age of 22 years (IQR:20–26), less educated, with only 32.7 % reporting secondary or higher level of education, and had a lower income, with 74.7 % earning a daily household income < $2 USD. Almost a third of women (28.7 %) perceived their partners were having concurrent partnerships. During the preceding six months, a higher proportion of women (30.7 %) than men reported experiencing physical threat either from their current partner (76.1 %) or another partner or other person including family members (23.9 %). Overall, 16 % of the women were HIV-infected. Over half (56.0 %) of the women had not used any contraceptive method prior to the index pregnancy.

**Table 1:** Baseline characteristics of study population, both pregnant women and their male partners (*N* = 488)

Characteristic		Women *N* = 300	Men *N* = 188
		n (%) median (IQR)	n (%) median (IQR)
Age (years)		22 (20,26)	29 (25,35)
Age group (years)			
	≤19	65 (21.7)	1 (0.5)
	20-24	140 (46.7)	33 (17.6)
	25-29	57 (19.0)	64 (34.0)
	30-34	27 (9.0)	41 (21.8)
	35-39	8 (2.7)	26 (13.8)
	≥40	3 (1.0)	23 (12.2)
Highest education level			
	Primary or lower	202 (67.3)	109 (58.0)
	Secondary (some or complete)	80 (26.7)	57 (30.3)
	Post secondary	18 (6.0)	22 (11.7)
Marital status			
	Monogamous	262 (87.3)	165 (87.8)
	Polygamous	25 (8.3)	17 (9.0)
	Unmarried (Single, widow or cohabiting)	13 (4.3)	6 (3.2)
Economic status			
	Daily household income ≥ $2 USD	76 (25.3)	113 (60.1)
Sexual partnerships			
	Self report of concurrency past 6 months	8 (2.7)	31 (17.0)
	Perceived partner concurrency	86 (28.7)	20 (10.6)
Physically threatened past 6 months			
	None	208 (69.3)	126 (67.0)
	Study partner	70 (23.3)	34 (18.1)
	Other partner or family member	22 (7.3)	28 (14.9)
Experienced forced sex			
	None	290 (96.7)	181 (96.3)
	Study partner	8 (2.7)	5 (2.7)
	Other partner or family member	2 (0.6)	2 (1.1)
HIV status			
	HIV positive	48 (16.0)	30 (16.0)
Number of previous pregnancies (or of partner)			
	None	51 (17.0)	28 (14.9)
	1	86 (28.7)	49 (26.1)
	≥2	163 (54.3)	111 (59.0)
Desire of more children			
	None	62 (20.7)	30 (16.0)
	Desire more children	238 (79.3)	158 (84.0)
Contraceptive use (or use by partner) prior to current pregnancy			
	None	168 (56.0)	128 (68.1)
	Hormonal	124 (41.3)	45 (24.0)
	Withdrawal, condom, sterilization	8 (2.7)	15 (8.0)

*IQR* interquartile range

### Baseline preferences on male partner HIV testing setting

Most participants preferred home-based (59.4 %) over ANC clinic-based (28.3 %) and VCT center-based (12.3 %) male partner HTC during pregnancy (Table 2). In addition, more men than women (68.1 vs. 54.0 %, p = 0.002) preferred home-based male partner HTC. Male partners were significantly more likely than pregnant women to prefer home-based HTC. After adjusting for partner HIV status, male partners remained significantly more likely than women to prefer HBHCT. Fewer men than women (19.2 % vs. 34.0 %, *p* < 0.001) preferred ANC clinic-based HTC. VCT center-based testing was the least preferred setting of male partner testing by both men (12.8 %) and women (12.0 %) and the difference was not statistically significant. The preferences did not vary by partner testing status.

**Table 2:** Differences in baseline preferences on setting of male partner HIV testing by gender

	Male		Female		0R	95 % CI	p-value
Home	128	(68.1)	162	(54.0)	1.81	1.24-2.66	0.002
Clinic	36	(19.2)	102	(34.0)	0.46	0.30-0.71	<0.001
VCT	24	(12.8)	36	(12.0)	1.07	0.62-1.86	0.796

### Correlates of preferred settings for male partner testing

Men were more likely to prefer HBHTC testing if they desired more children [odds ratio (OR) 3.47, 95 % confidence interval (CI): 1.53-7.89) (Table 3). This association remained significant after adjusting for partner HIV testing and randomization arm (adjusted OR 3.51, 95 % CI: 1.54-7.97, P = 0.003). Women who had primary or lower level of education were more likely than those with higher education to favor HBHCT for male partners and those who had a daily income < $2 USD were also more likely to prefer HBHCT for male partner testing. Women who perceived their partners as having concurrent partners were more likely to prefer HBHTC (OR = 2.35, 95 % CI: 1.39-3.99) and those who reported physical threat were also more likely to prefer HBHTC for male testing (OR1.44, 95 % CI: 1.06 to 1.95). HIV-infected women were more likely than HIV-uninfected women to favor male partner HBHTC (OR 2.35, 95 % CI: 1.20- 4.60).

**Table 3:** Correlates of preferred setting of male partner HIV testing at baseline by gender

Characteristic	Home				Clinic				VCT			
	Male		Female		Male		Female		Male		Female	
	OR	95 % CI	OR	95 % CI	OR	95 % CI	OR	95 % CI	OR	95 % CI	OR	95 % CI
Age ≥25 years	1.63	0.77-3.47	1.44	0.88-2.36	0.72	0.30-1.72	0.91	0.55--1.53	0.62	0.23-1.64	0.48	0.21-1.12
Education ≥ Secondary	1.04	0.56-1.92	O.47	0.29-0.77*	1.30	0.63-2.67	1.78	1.08--2.92*	0.65	0.27-1.58	1.56	0.77-3.14
Married	1.69	0.55-5.13	1.84	0.78-4.31	0.51	0.00-2.13	0.73	0.30--1.77	0.87	0.00-3.67	0.32	0.00-1.95
Income > $2 USD	0.73	0.39-1.35	0.53	0.31-0.90*	1.70	0.82-3.50	1.38	0.81--2.35	0.90	0.38-2.15	2.07	1.01-4.25*
Concurrency	1.41	0.59-3.27	6.19	0.75-50.9	0.60	0.20-1.76	0.00	0.00--0.91*	1.01	0.34-3.06	1.05	0.00-6.81
Perceived concurrency	2.00	0.64-6.26	2.35	1.39-3.99*	1.00	--	0.58	0.33-1.01	1.85	0.56-6.09	0.36	0.14-0.97*
Physically threat	1.29	0.93-1.79	1.44	1.06-1.95*	0.95	0.70-1.29	0.92	0.68-1.23	0.50	0.24-1.05	0.22	0.07-0.71*
Forced sex	1.43	0.37-5.54	1.22	0.49-3.01	0.63	0.10-3.82	0.70	0.24-2.06	0.89	0.15-5.24	1.20	0.37-3.87
HIV positive	0.92	0.40-2.12	2.35	1.20-4.60*	1.35	0.53-3.45	0.46	0.22-0.96*	0.73	0.20-2.64	0.63	0.21-1.86
≥1 previous pregnancy	1.03	0.89-1.19	1.08	0.95-1.23	0.94	0.79-1.13	0.95	0.83-1.10	1.03	0.85-1.24	0.92	0.74-1.14
Desire more children	3.47	1.53-7.89*	1.49	0.86-2.57	0.50	0.21-1.22	1.12	0.63-2.00	0.29	0.11-0.78*	0.36	0.17-0.75*
Contraceptive use	1.01	0.98-1.04	0.87	0.71-1.06	0.98	0.82-1.17	1.08	0.89-1.34	1.00	0.96-1.03	0.86	0.86-1.55

Comparison of Home versus Others, Clinic versus others and VCT versus others

OR odds ratio, CI confidence interval

*P < 0.05

### Changes in preferred settings for male partner testing

Overall, male and female participants were significantly more likely to prefer HBHTC for male testing at follow-up (71.0 %) compared to enrollment (59.2 %), (OR 1.98, 95 % CI: 1.43-2.78) irrespective of the study arm (Table 4). Men were twice as likely to favor male HBHTC during follow-up (80.9 %) compared to enrollment (68.1 %), (OR 2.20, 95 % CI: 1.27-3.94) (Table 4). Similarly, participating women were significantly more likely to favor male HBHTC at follow-up compared to baseline, (OR 1.89, 95 % CI: 1.26-2.89).

**Table 4:** Changes in preferred model of male partner HIV testing by gender^a^

	Male					Female				
Setting	Baseline	Follow-up	OR	95 % CI	p-value	Baseline	Follow-up	OR	95 % CI	p-value
Home	68.1 %	80.9 %	2.20	1.27-3.94	0.004**	54.0 %	65.3 %	1.89	1.26-2.89	0.001**
Clinic	19.5 %	11.9 %	0.61	0.38-0.98	0.039**	34.0 %	25.3 %	0.55	0.35-0.87	0.006**
VCT	13.0 %	7.6 %	0.50	0.21-1.12	0.068	12.2 %	9.5 %	0.72	0.39-1.31	0.258

^a^McNemar’s test OR odds ratio, CI confidence interval

**P < 0.05

Men were less likely to prefer ANC clinic-based or VCT center-based testing at follow-up. However, the decrease was not statistically significant. Women were significantly 45 % less likely at follow-up (25.3 %) than at enrollment (34.0 %) to favor ANC clinic-based male testing. Fewer women preferred VCT center-based testing at follow-up, although the difference was not statistically significant. Adjusting for partner testing status and study arm did not significantly alter the changes in preferences for male partner testing.

## Discussion

In this study, both pregnant women and their male partners preferred home-based HIV testing and counseling (HTC)  to the currently recommended antenatal (ANC) clinic setting or widely available VCT center-based testing. This suggests that higher HIV testing rates for male partners may be achieved through a home-based approach. Efforts to increase male partner involvement in PMTCT programs should consider offering women in ANC opportunities to have home visits for male partner HTC. Home-based male partner testing may influence PMTCT program effectiveness given some evidence that male partner involvement is associated with improved PMTCT uptake and infant HIV-free survival [1, 2, 7, 22].

We also found that more men than women preferred male partner HBHTC. This may have been because most men were enrolled and tested at home. However, this preference remained after adjusting for randomization arm and male partner HIV status. The high male partner preference for HBHTC compared to alternate settings is consistent with high uptake of male HTC reported in home-based HTC studies in South Africa [23] and Zambia [24, 25]. Men may prefer home testing due to the convenience, privacy and ease of access. In addition, with couples HBHTC mutual disclosure may reduce HIV-related stigma and increased partner support in preventing HIV acquisition by the HIV-uninfected partner and linkage to care for the HIV-infected partner or couple.

The other salient finding from this study was that desire for more children was substantially and significantly associated with preference for home-based male partner testing among male partners. This finding is important because home-based testing may provide an opportunity to protect couples against HIV acquisition in subsequent pregnancies and enhance PMTCT. Similarly, the majority of participating women and especially those who preferred home partner testing had a lower level of education and low daily household income. Therefore, a home-based approach may reach a majority of women and their partners in low-resource settings. It is possible that women with higher education level and income may have better negotiation skills for ANC clinic testing or have partners with similar or higher socioeconomic status who understand and support ANC clinic-based testing.

Concurrent and multiple partnerships increase the risk of HIV acquisition. In this study women were less likely to report concurrency and more likely to perceive concurrency than men and polygamy was low. The finding that pregnant women who reported perceived partner concurrency were more likely to favor home-based male partner testing suggests that such women were concerned about their partner’s HIV status and if in polygamous relationships would prefer HI testing of all partners at home. Specifically, they may have anticipated that a home-based approach was more likely to result in knowledge of their male partner’s HIV status. Women who reported physical threat were also more likely to favor home-based male partner testing most likely due to similar reasons in addition to possible reassurance of couple HTC and support. Therefore, home-based testing may reach women who consider themselves at greater risk of exposure to HIV from their partners and those who may need support for mutual disclosure as seen in other studies [21]. HIV-infected women were more likely to favor home-based male partner HTC possibly because such mothers may have known their HIV status earlier and needed support for couple HTC and mutual disclosure of results facilitated by home visits.

While ANC clinic-based testing remains the main setting of male partner testing, both women and men, but particularly the men, were less likely to favor ANC-based testing. This may explain the persistently low male partner and couple ANC testing rates seen in several studies despite the campaigns and motivation to make clinics male-friendly [7, 10, 13]. Additionally, very few pregnant women and few male partners preferred VCT-based male HTC. In other studies VCT testers were mostly single adults [12, 26], with few testing as couples. Thus, VCT -based testing may not encourage couples counseling and mutual disclosure of results. In our study, VCT-based testing was preferred by women who had higher income and considered themselves to be at a low risk of HIV acquisition (no perceived concurrency and no desire for additional children). This is contrary to a South African study which found that repeat VCT-based testers tended to be at greater risk of HIV, although it is important to note that these testers were predominantly single [26].

This study had several strengths. It was conducted in a low-resource setting with high HIV burden where male partner testing is low. Therefore, its findings can be generalized to similar settings with similar stable heterosexual partnerships. The three HTC options presented to participants in this study are the most commonly available modes of HTC in such settings, which also contributes to its generalizability. The study had some limitations. Specifically, we did not provide all of the different modes of HTC but only asked participants about their HTC preferences. Therefore, the study question captured intention to test rather than actual testing of male partners in the three different HTC models. We offered home-based testing to half of women and their partners and the other half were offered ANC clinic-based testing (no one was offered VCT center-based testing). This may have influenced participant responses and preferences. However, both arms of the study preferred home-based testing. Finally, the study was not designed to evaluate the reasons behind the preference of different settings for male partner HTC. Additional questions on the impact of this strategy may be assessed in long-term and qualitative studies.

## Conclusions

Pregnant women and their male partners were more likely to prefer a home-based approach for male partner HTC during pregnancy when given the options of home-based, ANC clinic-based or VCT center-based testing. To increase male partner testing, programs should provide pregnant women and their male partners with an opportunity for home visits for male partner testing during pregnancy. Future studies could evaluate this programmatic impact on PMTCT and other HIV prevention and treatment outcomes in the population.
